# High level of psychological stress in COVID-19 recovered individuals: role of copeptin as a potential biomarker

**DOI:** 10.3389/fpsyg.2023.1253396

**Published:** 2023-12-13

**Authors:** Phibakordor Lyngdoh Nonglait, Sri Venkata Madhu, Nishant Raizada, Amitesh Aggarwal, Rafat Ahmed, Mohammad Aslam

**Affiliations:** ^1^Department of Endocrinology, Centre for Diabetes Endocrinology and Metabolism, University College of Medical Sciences (University of Delhi) and GTB Hospital, New Delhi, India; ^2^Department of Medicine, University College of Medical Sciences (University of Delhi) and GTB Hospital, New Delhi, India; ^3^Department of Medical Biochemistry, University College of Medical Sciences (University of Delhi) and GTB Hospital, New Delhi, India

**Keywords:** COVID-19, cortisol, copeptin, stress, PSS-10

## Abstract

**Background:**

Study aimed to assess stress in COVID-19 recovered individuals using a validated questionnaire PSS-10 score and stress biomarkers – salivary cortisol and serum copeptin.

**Methods:**

A total of 83 subjects of which 54 subjects (66.3%) who were hospitalized were recruited 8–20 weeks following recovery from COVID-19. Stress was assessed by PSS-10 stress-scale after a mean duration of 14.5 weeks after recovery. Sixty-eight subjects (81.9%) had new or persistent symptoms after recovery. Subjects were divided into two groups on the basis of PSS score; mild stress (PSS:0–13) and moderate to severe stress (PSS:>14) and levels of biomarkers (serum copeptin, DHEAS and salivary cortisol) were compared in the two groups.

**Results:**

Forty-four subjects (53%) had moderate to severe stress and 39 subjects (47%) had mild stress. Subjects with post COVID symptoms had significantly higher stress levels as compared to subjects who were asymptomatic [15 vs. 9; *p* = 0.003]. Serum copeptin levels were significantly higher among subjects with moderate to severe stress as compared to those with mild stress [0.41 vs. 0.67 ng/mL; *p* = 0.031]. Subjects with moderate to severe stress had higher median salivary cortisol compared to subjects with mild stress [1.03 vs. 1.44 nmol/L; *p* = 0.448].

**Conclusion:**

Our study demonstrated moderate to severe stress in over half and some level of stress in nearly all COVID recovered individuals even after 3 months. Serum copeptin was found to be a useful biomarker to objectively measure stress in these subjects.

## 1 Introduction

The study proposes to assess stress in COVID-19 recovered individuals using perceived stress questionnaires and specific stress biomarkers.

COVID-19 has been reported to be associated with significant stress both during acute illness and after recovery (Mazza et al., 2020; Korompoki et al., 2021; Parker et al., 2021; Carola et al., 2022). This has been attributed to the direct effect of SARS-CoV-2 via direct neurotropic potential (Gregoriano et al., 2021; Raman et al., 2021) and cytokine dysregulation (Kappelmann et al., 2021) amplified by psychosocial stressors (Bornstein et al., 2020). SARS-CoV-2 is known to spread directly and indirectly into CNS where it has been shown to cause cytokine dysregulation and neuro-inflammation. Higher levels of cytokines – IL6 and IL1B and evidence of neuro-inflammation has been associated with post covid stress and neuro-psychiatric manifestations. Also, ICU survivors reflecting a greater cytokine response, have been shown to be more likely to develop neuropsychiatric disorders including PTSD compared to those not admitted to ICU. SARS-CoV-2 induced mitochondrial dysfunction, free radical generation and oxidative stress are also believed to destroy neurotrophic support thereby compromising the individuals response to stress. It has also been suggested that persistence of SARS-CoV-2 virus in the body even after recovery from acute illness could induce some levels of immune activation resulting in post COVID stress. Another mechanism predisposing to stress and stress related disorders include activation of HPA axis secondary to a damaged hippocampus and COVID related malnutrition and related tryptophan and vitamin D deficiency (Mohammadkhanizadeh and Nikbakht, 2021). In addition, a wide range of psychosocial stressors due to the pandemic are also believed to contribute to the COVID associated stress. These include; fear of the danger of COVID-19, worries about personal finances, grief after bereavement of close relatives and other effects of social isolation which affect family and social support and fear about access to medical care and crowded living conditions (Taylor et al., 2020; Mohammadkhanizadeh and Nikbakht, 2021).

PSS (Perceived stress score) is a validated questionnaire that gives information regarding perceived stress during the last 1 month. Based on their PSS scores, the level of stress can be classified as mild, moderate or severe. Perceived stress has been studied in subjects with chronic stress and 10 pm salivary cortisol correlated significantly with PSS and presumptive stressful life events scale – lifetime score (Siddiqui et al., 2015).

Psychological stress influences the homeostatic equilibrium of the body, through activation of the sympathetic nervous system and hypothalamic pituitary adrenal axis (HPA), by the release of corticotrophin-releasing hypothalamic factor (CRH) and arginine vasopressin (AVP) from the paraventricular nucleus of the hypothalamus, triggering cortisol secretion, which is considered a major contributor in stress response (Lightman, 2008; Belda et al., 2016). Salivary cortisol had been studied as a physiological marker of stress. However, it is an indirect measure of stress. The stress response of the HPA axis is complex and is modulated by other factors such as inflammation, sex steroids, adrenal sensitivity, cortisol binding, etc. (Hellhammer et al., 2009; Salzano et al., 2021). This poses a limitation and studies have demonstrated a correlation of merely 25% between perceived emotional stress variables and salivary cortisol levels (Campbell and Ehlert, 2012).

In chronic stress, it has been demonstrated that the hypothalamic activation of the pituitary changes from CRH hormone dominant to AVP dominant (Ma et al., 1997). AVP is derived from a larger precursor molecule along with two other peptides; neurophysin II and copeptin. Copeptin has been established to be a sensitive surrogate biomarker for AVP release as AVP is difficult to measure due to its instability and short half-life. Serum copeptin has been used as a biomarker for stress (Madhu et al., 2020). It has the advantage of lower serum volume, molecular stability, and availability of rapid and sensitive immunoassays which makes it an attractive biomarker (Martino and Arnaldi, 2021).

Most of the studies in COVID-19 recovered individuals assessed the psychiatric component following recovery and very few studies have measured stress directly and objectively. Assessment of stress using validated instruments and combining these measurements with objective stress biomarkers in COVID-19 recovered individuals will not only provide a comprehensive and objective assessment of stress but can also provide insights into the body’s response to COVID. This will help tailor support and treatment to facilitate rapid recovery of these individuals. In this study, we aimed to assess stress in COVID-19 recovered individuals using a validated questionnaire PSS-10 score and stress biomarkers – salivary cortisol and serum copeptin.

## 2 Methods

Assessment of stress was done using stress questionnaire PSS-10 and stress biomarkers- salivary cortisol and serum copeptin measurements in COVID-19 recovered individuals (*n* = 83).

### 2.1 Ethical clearance

The study was carried out as per guidelines of the Institutional Ethical Committee and written informed consent was obtained from all the study participants.

### 2.2 Sample size

A sample size of 60 subjects was required for PSS-10 assessment. It was based on; a standard deviation of 18.3 reported in previous study (Demerdash et al., 2021). Two sided significance level of 5 and 80% power of study were taken into account.

Similarly, based on serum copeptin, considering the SD of 8.6, with a relative marginal error of 10% on either side and *α* = 5%, a sample of 15 cases were required for copeptin to be used as a valid biomarker of stress.

### 2.3 Study design

A total of 83 COVID-19 recovered patients were enrolled in the study. These were patients who were positive for COVID-19 by RT-PCR/RAT. Patients with all grades of severity during their acute illness were included. Assessment of stress and sampling for all the biomarkers were done after a period of 8–20 weeks of recovery from COVID-19.

Recovery was defined as per the Indian Council of Medical Research criteria:

1. Mild cases: 10 days after symptoms onset and no fever for 3 days.
2. Moderate cases: 10 days after symptoms onset if fever resolved within 3 days and saturation maintained above 95% for next 4 days or if symptoms and oxygen requirement persisted beyond 3 days.
  Recovery was defined as resolution of symptoms with maintenance of oxygen saturation for 3 consecutive days.
3. Severe cases: Resolution of symptoms and negative RT-PCR.

Subjects were excluded if they were on steroids at the time of study, pregnant or lactating or having chronic active infection including tuberculosis, mucormycosis.

After enrolment, details of hospitalization and clinical examination were recorded in a predesigned proforma. PSS-10 questionnaire was administered by the investigator for assessment of stress. Based on their PSS scores, the subjects were divided into mild (PSS: 0–13), moderate (PSS: 14–26), and severe stress (PSS: 27–40) groups.

All the subjects were a provided a salivette (saliva collection tube that contains cotton swab) for salivary cortisol collection at home at 10 pm after ensuring good oral hygiene. They were instructed to chew the cotton swab for at least 60 s for an adequate sample. Participants were advised to abstain from brushing their teeth, smoking, eating, or drinking 2 h prior to sample collection. They were told to bring the salivary sample the next morning and report after an 8-h overnight fast for further biochemical testing.

Venous blood samples were collected at 8–9 AM for hematological, biochemical tests, inflammatory markers and stress biomarkers. All precautions necessary for sample collection were taken. Samples for salivary cortisol and copeptin were centrifuged and stored at −80°C till further analysis.

Tests done included:

- Complete haemogram, fasting plasma glucose, HbA1C, kidney function test, Liver function test and lipid profile.
- Serum CRP, IL-6, ferritin, serum copeptin, DHEAS and salivary cortisol.

### 2.4 Biochemical analysis

Complete hematological (HORIBA, USA) and biochemical investigations (Randox Laboratories, UK) were done by commercially available kits on fully automated analyzers.

IL-6 and ferritin measurements were done by CLIA method on ACCESS II auto (Beckman coulter, USA) using system packs. DHEAS was measured by CLIA method on IMMULITE^®^ 2000 XPi (SIEMENS). Salivary cortisol levels were measured by Radioimmunoassay (Beckman Coulter, Czech Republic). Serum copeptin and CRP were measured by ELISA (RayBiotech, USA).

The inter and intra assay coefficients of variation for salivary cortisol was <14.5 and 9.5%, respectively. Inter and intra assay coefficients of variation for rest of the parameter were < 10%.

### 2.5 Statistical analysis

Categorical data were expressed as numbers (%), continuous data as mean ± SD (95% CI) when normally distributed and median (interquartile range [IQR]) when distribution was not normal. Tests for normality were done for continuous variables and non-Gaussian data was appropriately transformed to normalize it. Categorical data were compared between groups using parametric (chi-square test/ Fischer exact test) and non-parametric tests (Mann–Whitney tests) as appropriate. Continuous data were compared between two groups using independent sample *t*-test. A two-tailed *p* value of <0.05 was considered statistically significant. The analysis was performed on SPSS version 23.0.

## 3 Results

A total of 83 participants with a mean age of 45.31 ± 12.1 years were included in the study of whom 50 (60.2%) were male and 33 (39.8%) were female. The most common symptoms reported by participants after recovering from COVID was fatigue (62%), followed by breathlessness (50.6%), palpitations (38.5%), brain fog (27.7%), insomnia (21.6%), and chest discomfort (20.4%). Subjects also reported symptoms of irritability (21.6%), and anxiety (7.2%).

Twenty-eight patients (33.7%) had underlying comorbidities, of which hypertension and diabetes mellitus was most commonly reported as shown in Table 1. Seven patients (8.4%) had coexisting hypertension and diabetes mellitus. Of the 16 patients with a history of hypertension, 13 were on medications and the rest were not on any treatment. Among the 16 patients with underlying diabetes, 4 reported hyperglycaemia at the time of hospitalization for acute COVID. All of these subjects were either on insulin or oral hypoglycaemic agents.

**Table 1:** Demographic, details of hospitalization and anthropometric details of subjects stratified as per stress severity by PSS score.

Parameter	Total	Mild stress (PSS score 0–13)	Moderate to severe stress (PSS score 14–40)	Odds ratio (CI)	*p* value
Subjects (%)	83	39 (46.9%)	44 (53.1%)		
Age (Yrs)	45.3 ± 12.1	44.7 ± 13.6	45.82 ± 10.8		0.69
Female	33	15	18	0.90 (0.37–2.18)	0.49
Disease severity					
Mild covid	33	17	16	0.74 (0.31–1.78)	0.65
Moderate covid	9	4	5	1.12 (0.28–4.5)	1.0
Severe covid	41	18	23	1.27 (0.54–3.03)	0.66
Hospitalization	54	25	29	1.08 (0.44–2.67)	1.0
ICU stay	13	5	8	1.51 (0.45–5.07)	0.56
Supplemental oxygen	51	22	29	1.49 (0.61–3.63)	0.49
Steroid use	50	23	27	1.11 (0.45–2.66)	1.0
Vaccination	18	9	9	0.85 (0.30–2.47)	0.79
Time since recovery, weeks	14.5 ± 3.4	15.5 ± 3.4	14.09 ± 3.4		0.21
Post covid symptoms	68	27	41	6.07 (1.56–23.55)	0.008
Comorbidities					
Hypertension	16	7	9		1.0
Diabetes mellitus	16	6	10		0.42
Ischemic heart disease	2	0	2		0.49
COPD/bronchial asthma	3	1	2		0.49
Dyslipedemia	2	0	2		0.49
Anthropometry					
BMI (kg/m^2^)	26.06	26.14 ± 3.75	25.98 ± 3.72		0.85
Waist circumference (cm)	96.5 ± 11.2	95.67 ± 11.7	97.31 ± 10.9		0.51

Table 1 represents the demographic and anthropometric details of subjects stratified by severity of stress into mild stress and moderate to severe stress by PSS. As can be seen from the table, 46.9% of COVID recovered subjects had mild stress while 53.1% of them had moderate/severe stress (48.2% moderate and 4.8% severe) based on PSS scores. There was a significant increase in the post COVID symptoms (*p* = 0.008) in subjects with moderate/severe stress as compared to those with mild stress. There was no significant difference in any of the other parameters between subjects with mild stress when compared with those with moderate/severe stress.

**Figure 1:**
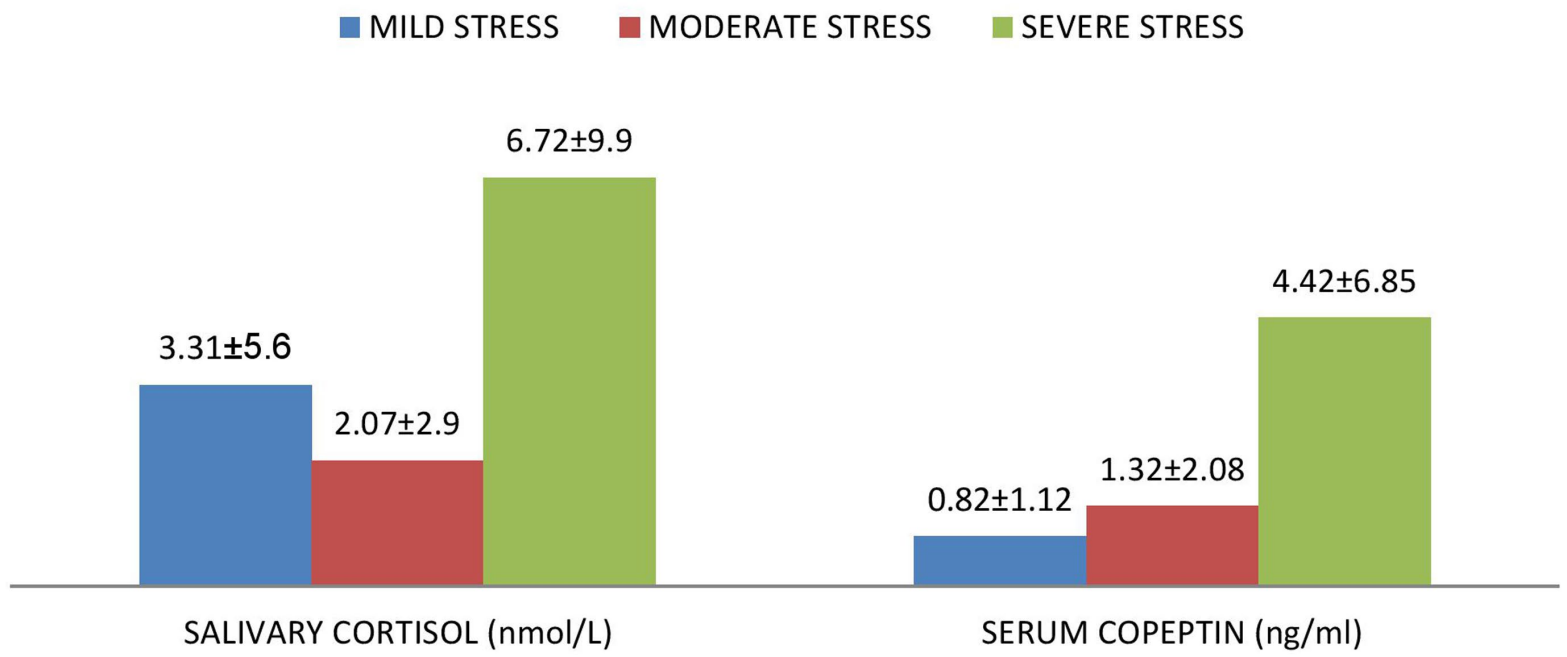
Mean ± SD of serum copeptin and salivary cortisol according to the various levels of stress among the subjects.

**Figure 2:**
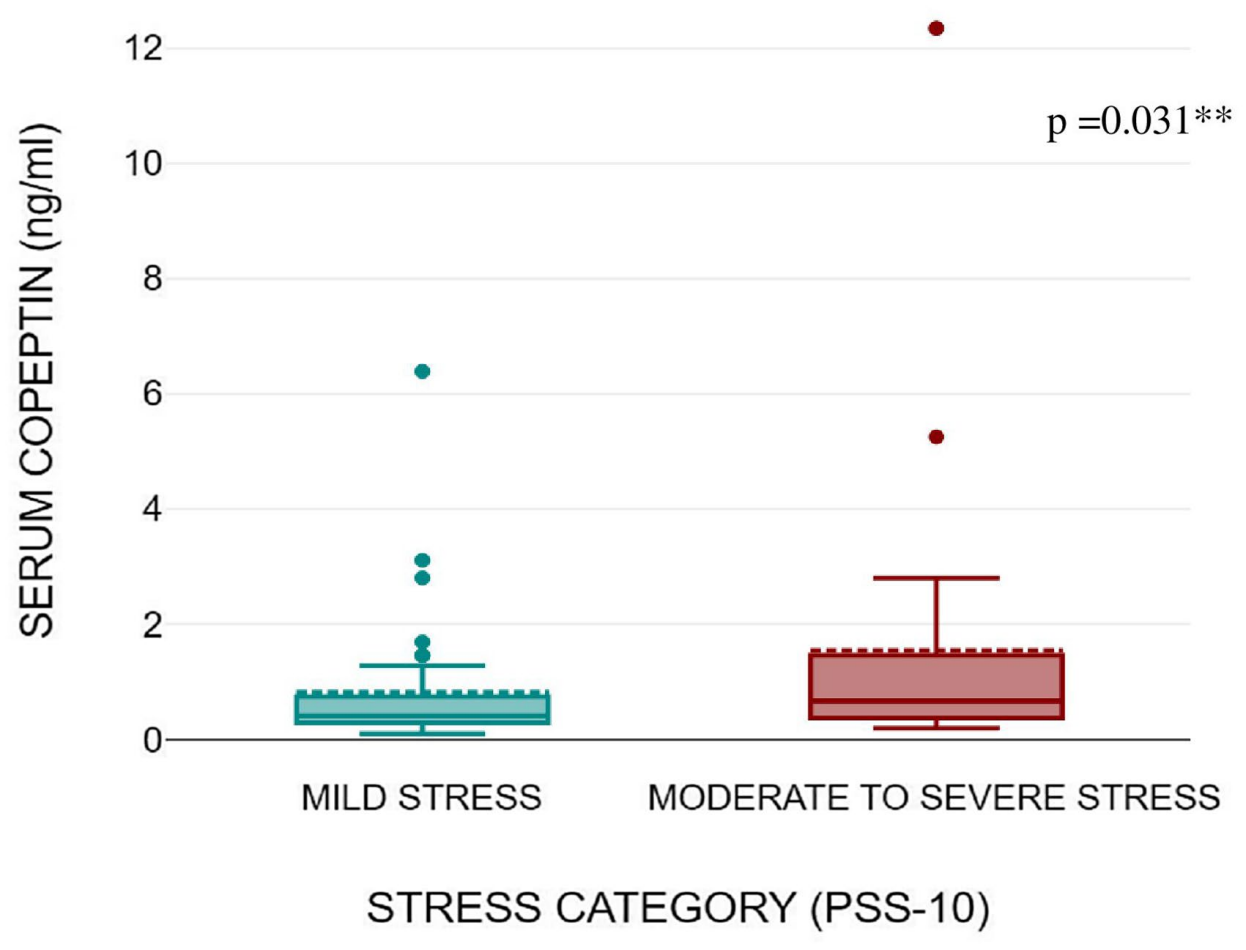
Box plot showing serum copeptin levels as per stress severity (mild stress and moderate to severe stress) among the subjects.

**Figure 3:**
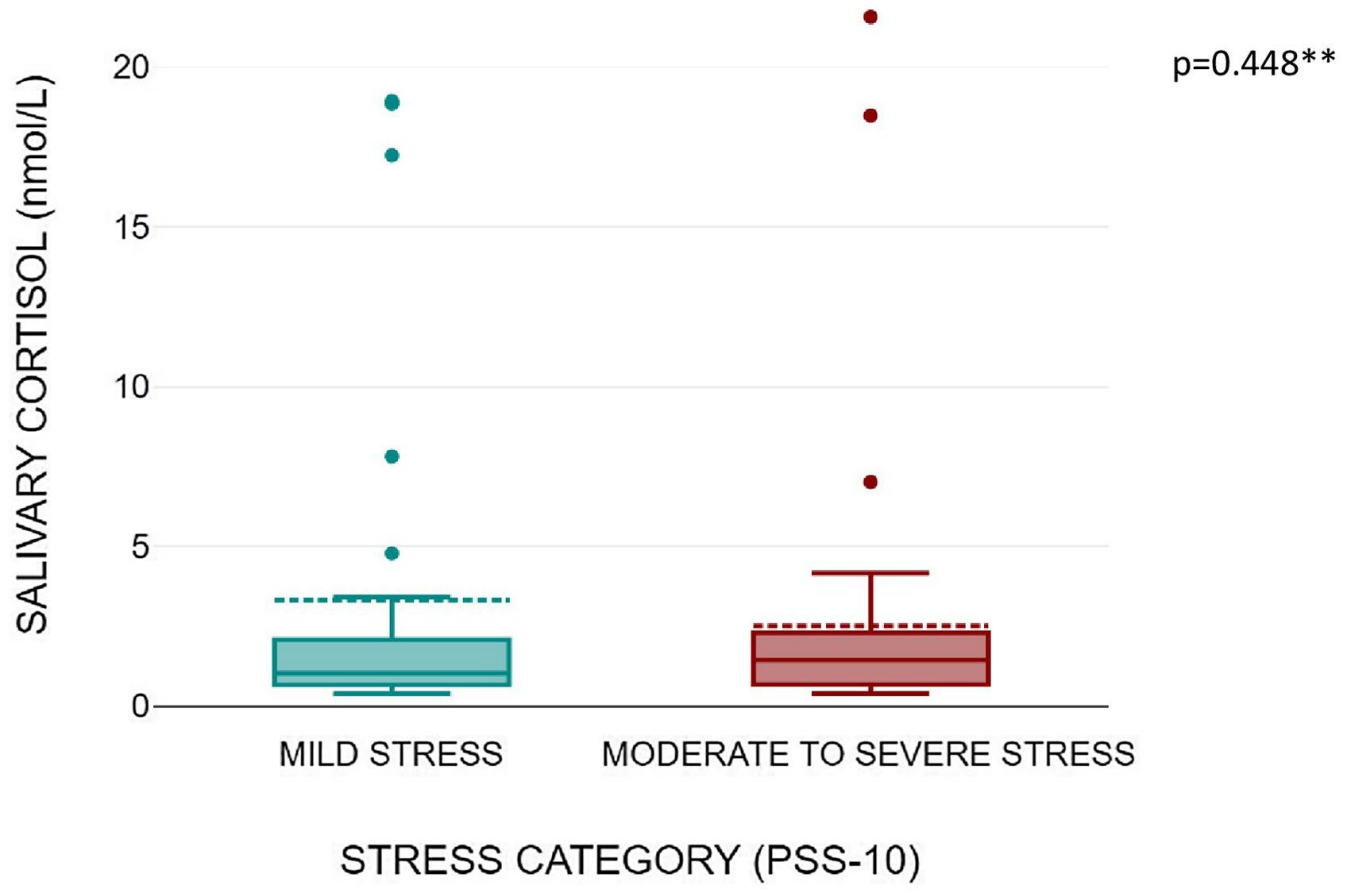
Box plot showing salivary cortisol levels as per stress severity (mild stress and moderate to severe stress among the subjects).

Figures 1, 2 shows that serum copeptin levels were significantly higher (*p* = 0.031) in COVID recovered patients with moderate /severe stress as compared to those with mild stress. However, no significant difference was observed between these two groups for median salivary cortisol levels by Mann Whitney *U* test (*p* = 0.448) as shown in Figure 3.

Subjects with elevated CRP had a higher median serum copeptin compared to subjects with normal CRP as assessed by Independent samples Median test (*p* = 0.007) (Table 2). There was no significant association between elevated IL-6 and ferritin with any of the parameters of stress.

**Table 2:** Difference in salivary sortisol and serum copeptin among the 2 groups (group 1: normal CRP, group 2: elevated CRP).

Parameter		Normal CRP (*n* = 44)	Elevated CRP (n = 36)	*p*-value
Serum copeptin (ng/ml)	Median (IQR)	0.4 (0.31–0.80)	0.72 (0.39–1.14)	0.007
Salivary cortisol (nmol/L)	Median (IQR)	1.44 (0.68–2.3)	1.175 (0.61–2.14)	0.13

** Independent samples Median test.

**Figure 4:**
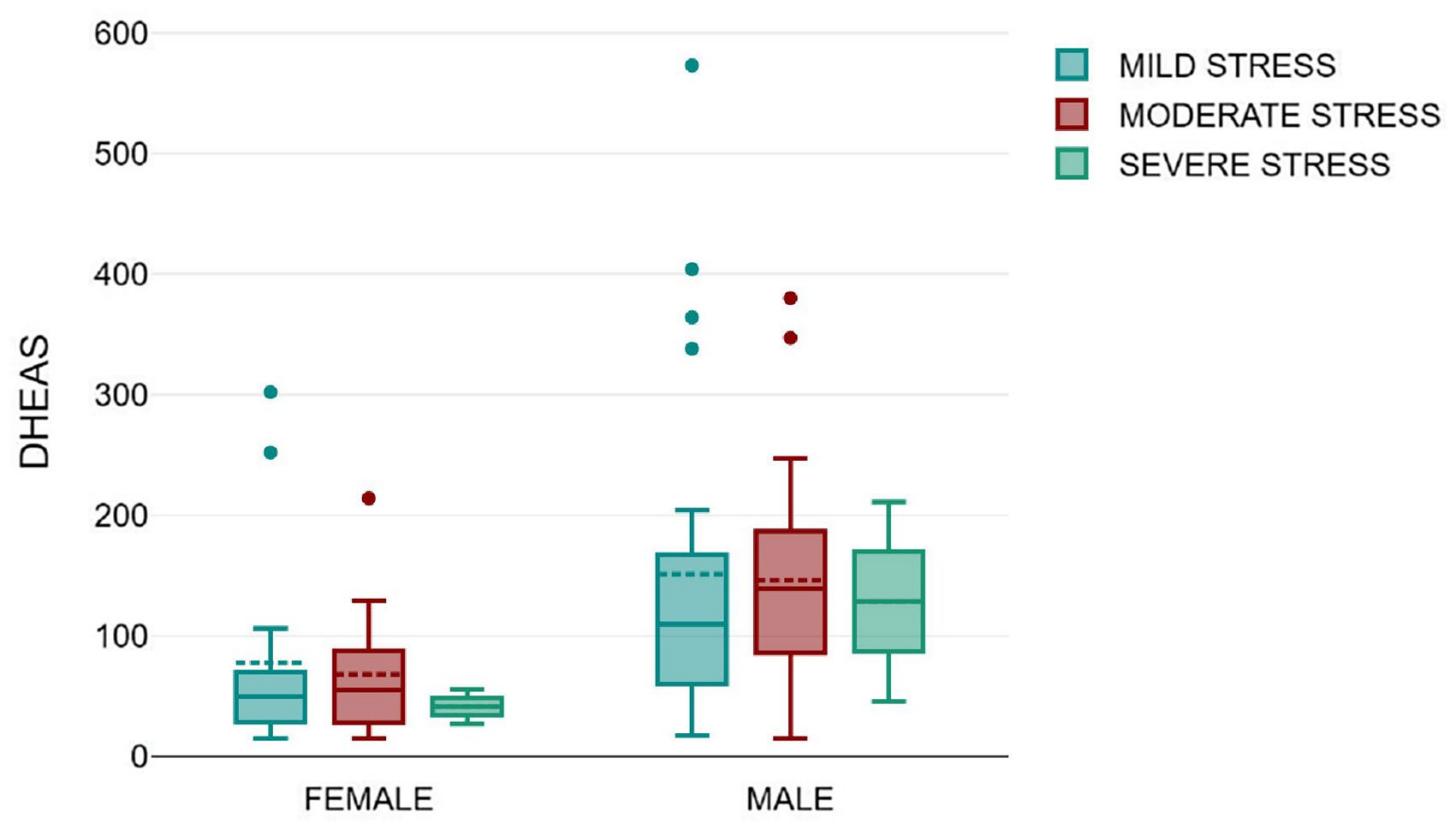
Mean DHEAS (mcg/dl) among males and females as per stress severity by PSS.

We also observed that subjects with severe stress had lower mean DHEAS as compared to subjects with mild or moderate stress as depicted in Figure 4, although this was not statistically significant by independent t tests (*p* = 0.634).

## 4 Discussion

Our study found that over half of the subjects who had recovered from COVID-19 illness displayed moderate or severe stress based on PSS score even at 8–20 weeks after recovery while all of the remaining subjects showed evidence of mild stress. Serum copeptin levels, which served as a stress biomarker, were significantly higher in subjects with moderate/severe stress when compared with those of mild stress. On the other hand, salivary cortisol levels were not found to be different between the two groups. Copeptin levels were higher in those with higher CRP reflecting greater inflammation.

Very few studies have been done to directly measure stress in COVID-19-recovered individuals. Even these focussed on hospitalized subjects and those with mild or asymptomatic disease were not well represented. In contrast our study included individuals with all grades of severity and also used objective biomarkers – serum copeptin and salivary cortisol to make the measurement of stress more robust. To the best of our knowledge, no study has been done to explore serum copeptin as a stress marker in COVID recovered individuals.

Our study found that over half of our subjects had moderate to severe stress and the remaining mild stress based on their PSS-10 scores. This was higher than 2 earlier studies in COVID recovered individuals reported from Italy and USA. One of these studies (Carola et al., 2022) also using PSS-10 stress scale in those who had recovered from severe COVID reported moderate or severe stress in 30% of them and mild stress in 70% of them 1–3 months after recovery. However, they did not measure serum copeptin levels or any other biomarker. The retrospective nature of our analysis and the larger window of assessment of 8–12 weeks post recovery in our study could have contributed to the differences in prevalence of stress in both these studies. The other study (Parker et al., 2021) reported that only 25% of those who had recovered from COVID experienced mild or moderate stress at 2 weeks of follow-up (Parker et al., 2021). The lower stress levels in this study may be explained by the fact that 16% of the subjects had received psychiatric consultation and 11 (18.5%) subjects were already on psychotropic medications which may have reduced the stress levels at follow-up.

It has been proposed that studies evaluating stress particularly in relation to COVID-19 should focus on the combined use of psychological and biomarker testing to increase accuracy (Moayed et al., 2021). PSS-10 score is a well validated instrument to measure stress as perceived by the individual over the previous 1 month (Siddiqui et al., 2015). Higher PSS-10 scores clearly point to significant levels of perceived stress in COVID recovered individuals during the 4 weeks preceding their enrolment in the study. Addition of Copeptin, a stress biomarker, helps confirm the presence of stress objectively. It can provide useful information on the severity of stress and when measured at different timepoints can help trace the recovery from stress.

We have explored the same by measuring salivary cortisol and serum copeptin as objective biomarkers of stress in addition to the PSS-10 questionnaire.

We found that serum copeptin levels were significantly higher in those with moderate and severe stress as compared to those with mild stress following recovery from COVID. Demerdash and co-workers studied stress in healthcare workers involved in the care of COVID-19 by measuring stress by perceived stress score (PSS) questionnaire and copeptin (Demerdash et al., 2021). The study revealed that baseline copeptin levels pre-quarantine were significantly increased and positively correlated with high stress. A decline in serum copeptin and PSS was observed following a decrease in stress post-quarantine (Demerdash et al., 2021). The role of copeptin as a stress biomarker in subjects who had recovered from COVID has not been explored so far.

Copeptin, a 39-amino acid glycopeptide, is a C-terminal part of the precursor pre-provasopressin. Activation of the AVP system stimulates copeptin secretion into the circulation from the posterior pituitary gland in equimolar amounts with AVP. Copeptin therefore directly reflects AVP secretion and has been used as a surrogate biomarker of AVP secretion (Morgenthaler et al., 2008). Earlier studies have shown that individuals with psychological stress display increased AVP secretion from neurons of hypothalamus (Pasquali et al., 1996; Bao et al., 2014). Also, clinical and experimental studies also show that under chronic stress conditions, AVP plays a more dynamic role in activation of HPA axis than CRH (De Goeij et al., 1992; Bao et al., 2014). AVP is believed to regulate the HPA axis in chronic stress conditions by modulating the neuroendocrine responses involved in coping with stress (Madhu et al., 2020). Further, AVP is believed to be important in facilitating and maintaining the hyper responsiveness of pituitary corticotrophs in the face of chronically elevated circulating cortisol levels associated with chronic stress (Aguilera and Rabadan-Diehl, 2000). Therefore, AVP appears to be critical in the HPA axis adaption to chronic stress and serum copeptin can serve as a valuable biomarker of chronic stress. Studies have confirmed the usefulness of Copeptin in patients with chronic psychological stress (Ma et al., 1997; Bao et al., 2014; Madhu et al., 2020; Martino and Arnaldi, 2021).

We observed a higher median salivary cortisol among subjects with moderate and high stress as compared to mild stress by PSS. However, we did not demonstrate any statistically significant difference between these two groups. Salivary cortisol has been studied as a physiological marker of stress. Perceived stress has been studied in subjects of chronic stress and 10 pm salivary cortisol correlated significantly with PSS score and presumptive stressful life events scale – lifetime score (Siddiqui et al., 2015). The correlation between perceived emotional stress and salivary cortisol has been demonstrated only in 25% of the studies earlier (Campbell and Ehlert, 2012). This is because salivary cortisol is an indirect measure of stress and the stress response of the HPA axis is complex and is modulated by many other factors such as inflammation, sex steroids, adrenal sensitivity, cortisol binding, etc. (Hellhammer et al., 2009). Our subjects were exposed to exogenous steroids, some of them for long periods which may have modulated the HPA axis and explained the lack of statistically significant difference in salivary cortisol levels between the two groups. Also, the inflammatory changes post-COVID may have masked the effects of stress on salivary cortisol as nearly half of our subjects had evidence of persistent inflammation. While there is no study in post COVID subjects that assessed salivary cortisol, Deneva et al. (2022) examined salivary cortisol, alpha amylase and Chromogranin A in adult hospitalized patients with COVID and demonstrated significantly high levels of these biomarkers in them.

Perceived stress as measured by PSS questionnaire was found to be significantly associated with post-COVID symptoms. However, salivary cortisol or serum copeptin did not have any such association. Most of the symptoms reported post-COVID are believed to be due to many factors of which stress has been found to have a significant association. Our study was able to demonstrate that stress even after recovery is associated with post –COVID symptoms which indicates that psychological factors may be linked to persistent symptoms after COVID. Similar observations were reported in a large cohort study where subjects with higher perceived stress prior to illness had a relative risk of 1.46 (95% CI, 1.18–1.81) for post-COVID symptoms (Wang et al., 2022).

Nearly half and over a fifth of subjects had high CRP and high IL-6 levels, respectively at 8–20 weeks post-COVID. Only 3 subjects with high ferritin levels were observed and these subjects also had elevated CRP and IL-6 levels. The persistence of inflammation has been proposed as a putative mechanism for symptoms after COVID recovery (Yong, 2021). We observed that subjects with elevated CRP had significantly higher serum copeptin as compared to those with normal CRP levels. However, there was no difference in salivary cortisol levels or PSS score in those with higher CRP. Also, there was no association of elevated IL-6 or ferritin with any of the markers of stress.

## 5 Limitations

Our study had certain limitations. Firstly, stress assessment parameters were not measured at baseline which would have provided information on stress levels at the onset of acute COVID-19 illness. This would have allowed a more definite conclusion on whether the high levels of stress being reported in COVID recovered individuals were due to COVID or reflected pre-existing stress. Secondly, we did not include a control group of individuals who were not affected by COVID. This could have informed us of the prevailing stress in the community at that time. Finally, we did not follow up our patients which could have helped ascertain the time period for recovery from stress.

## 6 Conclusion

In conclusion, our study demonstrated moderate to severe stress in over half and some level of stress in nearly all COVID recovered individuals even after 3 months. Serum copeptin was found to be a useful biomarker to objectively measure chronic stress in these subjects. Future prospective studies with longer follow up should be done to understand the time taken for stress to fully recover.

## Data availability statement

The raw data supporting the conclusions of this article will be made available by the authors, without undue reservation.

## Ethics statement

The studies involving humans were approved by Institutional Ethics Commitee – UCMS Delhi. The studies were conducted in accordance with the local legislation and institutional requirements. The participants provided their written informed consent to participate in this study.

## Author contributions

PN: Data curation, Formal analysis, Investigation, Writing – original draft, Writing – review & editing, Methodology. SM: Conceptualization, Methodology, Supervision, Visualization, Writing – original draft, Writing – review & editing. NR: Conceptualization, Formal analysis, Investigation, Methodology, Supervision, Writing – review & editing. AA: Writing – review & editing. RA: Writing – review & editing. MA: Data curation, Writing – review & editing.

## References

[ref1] Aguilera G. Rabadan-Diehl C. (2000). Vasopressinergic regulation of the hypothalamic–pituitary–adrenal axis: implications for stress adaptation. Regul. Pept. 96, 23–29. doi: 10.1016/S0167-0115(00)00196-8, PMID: 11102648

[ref2] Bao L.-L. Jiang W.-Q. Sun F.-J. Wang D.-X. Pan Y.-J. Song Z.-X.. (2014). The influence of psychological stress on arginine vasopressin concentration in the human plasma and cerebrospinal fluid. Neuropeptides 48, 361–369. doi: 10.1016/j.npep.2014.09.006 25454843

[ref3] Belda X. Nadal R. Armario A. (2016). Critical features of acute stress-induced cross-sensitization identified through the hypothalamic-pituitary-adrenal axis output. Sci. Rep. 6:31244. doi: 10.1038/srep31244, PMID: 27511270 PMC4980629

[ref4] Bornstein S. R. Dalan R. Hopkins D. Mingrone G. Boehm B. O. (2020). Endocrine and metabolic link to coronavirus infection. Nat. Rev. Endocrinol. 16, 297–298. doi: 10.1038/s41574-020-0353-9 32242089 PMC7113912

[ref5] Campbell J. Ehlert U. (2012). Acute psychosocial stress: does the emotional stress response correspond with physiological responses? Psychoneuroendocrinology 37, 1111–1134. doi: 10.1016/j.psyneuen.2011.12.010, PMID: 22260938

[ref6] Carola V. Vincenzo C. Morale C. Pelli M. Rocco M. Nicolais G. (2022). Psychological health in COVID-19 patients after discharge from an intensive care unit. Front. Public Health 10:951136. doi: 10.3389/fpubh.2022.951136 36033791 PMC9411785

[ref7] De Goeij D. Binnekade R. Tilders F. (1992). Chronic stress enhances vasopressin but not corticotropin-releasing factor secretion during hypoglycemia. Am. J. Physiol. Endocrinol. Metabolism 263, E394–E399. doi: 10.1152/ajpendo.1992.263.2.E394 1325125

[ref8] Demerdash H. M. Omar E. Arida E. (2021). Evaluation of copeptin and psychological stress among healthcare providers during COVID-19 pandemic. Egypt. J. Anaesth. 37, 227–233. doi: 10.1080/11101849.2021.1925442

[ref9] Deneva T. Ianakiev Y. Boykinova O. (2022). Salivary mental stress biomarkers in COVID-19 patients. Front. Med. 9:999215. doi: 10.3389/fmed.2022.999215 PMC9666483 36405600

[ref10] Gregoriano C. Molitor A. Haag E. Kutz A. Koch D. Haubitz S.. (2021). Activation of vasopressin system during COVID-19 is associated with adverse clinical outcomes: an observational study. J. Endocr. Soc. 5:bvab045. doi: 10.1210/jendso/bvab045, PMID: 34056499 PMC7989362

[ref12] Hellhammer D. H. Wüst S. Kudielka B. M. (2009). Salivary cortisol as a biomarker in stress research. Psychoneuroendocrinology 34, 163–171. doi: 10.1016/j.psyneuen.2008.10.026 19095358

[ref13] Kappelmann N. Dantzer R. Khandaker G. M. (2021). Interleukin-6 as potential mediator of long-term neuropsychiatric symptoms of COVID-19. Psychoneuroendocrinology 131:105295. doi: 10.1016/j.psyneuen.2021.105295, PMID: 34119855 PMC8172271

[ref14] Korompoki E. Gavriatopoulou M. Hicklen R. S. Ntanasis-Stathopoulos I. Kastritis E. Fotiou D.. (2021). Epidemiology and organ specific sequelae of post-acute COVID19: a narrative review. J. Infect. 83, 1–16. doi: 10.1016/j.jinf.2021.05.004, PMID: 33992686 PMC8118709

[ref16] Lightman S. L. (2008). The neuroendocrinology of stress: a never ending story. J. Neuroendocrinol. 20, 880–884. doi: 10.1111/j.1365-2826.2008.01711.x, PMID: 18601712

[ref17] Ma X. M. Levy A. Lightman S. L. (1997). Emergence of an isolated arginine vasopressin (AVP) response to stress after repeated restraint: a study of both AVP and corticotropin-releasing hormone messenger ribonucleic acid (RNA) and heteronuclear RNA. Endocrinology 138, 4351–4357. doi: 10.1210/endo.138.10.5446 9322950

[ref18] Madhu S. V. Aslam M. Siddiqui A. A. Goyal S. Mishra B. K. (2020). Association of copeptin with sense of coherence in individuals with varying degrees of glucose intolerance. Psychosom. Med. 82, 181–186. doi: 10.1097/PSY.0000000000000768 31738318

[ref19] Martino M. Arnaldi G. (2021). Copeptin and stress. Endocrine 2, 384–404. doi: 10.3390/endocrines2040035

[ref20] Mazza M. G. De Lorenzo R. Conte C. Poletti S. Vai B. Bollettini I.. (2020). Anxiety and depression in COVID-19 survivors: role of inflammatory and clinical predictors. Brain Behav. Immun. 89, 594–600. doi: 10.1016/j.bbi.2020.07.037, PMID: 32738287 PMC7390748

[ref21] Moayed M. S. Vahedian-Azimi A. Mirmomeni G. Rahimi-Bashar F. Goharimoghadam K. Pourhoseingholi M. A.. (2021). Depression, anxiety, and stress among patients with COVID-19: a cross-sectional study. Adv. Exp. Med. Biol. 1321, 229–236. doi: 10.1007/978-3-030-59261-5_19 33656727

[ref22] Mohammadkhanizadeh A. Nikbakht F. (2021). Investigating the potential mechanisms of depression induced-by COVID-19 infection in patients. J. Clin. Neurosci. 91, 283–287. doi: 10.1016/j.jocn.2021.07.023, PMID: 34373041 PMC8289699

[ref23] Morgenthaler N. G. Struck J. Jochberger S. Dünser M. W. (2008). Copeptin: clinical use of a new biomarker. Trends Endocrinol Metab 19, 43–49. doi: 10.1016/j.tem.2007.11.001 18291667

[ref24] Parker C. Shalev D. Hsu I. Shenoy A. Cheung S. Nash S.. (2021). Depression, anxiety, and acute stress disorder among patients hospitalized with COVID-19: a prospective cohort study. J. Acad. Consult. Liaison Psychiatry 62, 211–219. doi: 10.1016/j.psym.2020.10.001, PMID: 33198962 PMC7546958

[ref25] Pasquali R. Anconetani B. Chattat R. Biscotti M. Spinucci G. Casimirri F.. (1996). Hypothalamic-pituitary-adrenal axis activity and its relationship to the autonomic nervous system in women with visceral and subcutaneous obesity: effects of the corticotropin-releasing factor/arginine-vasopressin test and of stress. Metabolism Clin. Exp. 45, 351–356. doi: 10.1016/S0026-0495(96)90290-5, PMID: 8606643

[ref26] Raman B. Cassar M. P. Tunnicliffe E. M. Filippini N. Griffanti L. Alfaro-Almagro F.. (2021). Medium-term effects of SARS-CoV-2 infection on multiple vital organs, exercise capacity, cognition, quality of life and mental health, post-hospital discharge. EClinicalMedicine 31:100683. doi: 10.1016/j.eclinm.2020.100683, PMID: 33490928 PMC7808914

[ref27] Salzano C. Saracino G. Cardillo G. (2021). Possible adrenal involvement in long COVID syndrome. Medicina 57:1087. doi: 10.3390/medicina57101087, PMID: 34684123 PMC8537520

[ref28] Siddiqui A. Madhu S. V. Sharma S. B. Desai N. G. (2015). Endocrine stress responses and risk of type 2 diabetes mellitus. Stress 18, 498–506. doi: 10.3109/10253890.2015.1067677 26303379

[ref29] Taylor S. Landry C. A. Paluszek M. M. Fergus T. A. McKay D. Asmundson G. J. G. (2020). COVID stress syndrome: concept, structure, and correlates. Depress. Anxiety 37, 706–714. doi: 10.1002/da.23071, PMID: 32627255 PMC7362150

[ref30] Wang S. Quan L. Chavarro J. E. Slopen N. Kubzansky L. D. Koenen K. C.. (2022). Associations of depression, anxiety, worry, perceived stress, and loneliness prior to infection with risk of post–COVID-19 conditions. JAMA Psychiatry 79, 1081–1091. doi: 10.1001/jamapsychiatry.2022.2640, PMID: 36069885 PMC9453634

[ref31] Yong S. J. (2021). Long COVID or post-COVID-19 syndrome: putative pathophysiology, risk factors, and treatments. Infect. Dis. 53, 737–754. doi: 10.1080/23744235.2021.1924397, PMID: 34024217 PMC8146298

